# Tracking of lipids in schoolchildren: a four-year follow-up, population-based study in Sousse (Tunisia)

**Published:** 2010-02

**Authors:** I Harrabi, J Maatoug, R Gaha, H Ghannem, K Limam, AS Essoussi

**Affiliations:** Service d’Epidémiologie et des Statistiques Médicales, CHUF Hached, Sousse, Tunisia; Service d’Epidémiologie et des Statistiques Médicales, CHU F Hached, Sousse, Tunisia; Service d’Epidémiologie et des Statistiques Médicales, CHUF Hached, Sousse, Tunisia; Service d’Epidémiologie et des Statistiques Médicales, CHUF Hached, Sousse, Tunisia; Département de Biochimie , Faculté de Médecine de Sousse, Sousse, Tunisia; Service de Pédiatrie, CHU F Hached, Sousse, Tunisia

**Keywords:** lipids, tracking, adolescent, epidemiology

## Abstract

**Objective:**

Dyslipidaemia, which is now seen as one of the most important cardiovascular risk factors, is becoming more common in the younger population. The aim of this study was to assess the efficacy of tracking serum lipid levels over a four-year period in an urban population of schoolchildren.

**Methods:**

The study began in 1999 with a cohort of 789 schoolchildren. Four years later this group was resurveyed and a further 452 adolescent were recruited to the study.

**Results:**

The percentages of boys who were initially in the extreme quartile for total cholesterol (TC), low-density lipoprotein (LDL) cholesterol and triglycerides were 42.5, 54.8 and 40.4%, respectively. Similarly, the percentages of girls in the extreme quartile were 62.7, 53.8 and 38.2%. Four years later, both the boys and girls were still in the extreme quartile for these parameters. Therefore, the best predictor of follow-up level for each of the serum lipoprotein cholesterol fractions was the corresponding baseline level. Interestingly, the next best predictor in most of the groups was change in body mass index (ΔBMI) and smoking status.

**Conclusion:**

Prevention of coronary heart diseases in adults must begin early on in childhood, and should be driven by health education towards achieving a healthy lifestyle.

## Summary

The World Health Organisation (WHO) has helped avert 16.7 million deaths due to cardiovascular diseases by promoting healthy diet and lifestyle.^1^ Cardiovascular disease is the major cause of morbidity and mortality in most industrialised countries.^2^ Tunisia is now facing the phenomenon of an epidemiological transition, with an increase in chronic non-communicable diseases, particularly cardiovascular disease.^3^ Dyslipidaemia is seen as the major cardiovascular risk factor and it is becoming more prevalent among the young population.^4^,^5^ It has been established that atherosclerosis is a process that starts in childhood.^6^-^9^

The Bogalusa heart study identified a strong association of total cholesterol and low-density lipoprotein cholesterol with cholesterol found in the aortic and coronary vessels at post mortem.^10^,^11^ The tendency for an individual to maintain his/her level of disease risk factor relative to his/her peers through time has important implications in terms of determination of causality of disease, as well as for the prevention of future related morbidity and mortality.^12^ Several longitudinal studies have reported significant tracking of serum lipid levels from childhood to adulthood.^13^-^15^ In Tunisia there have not been any longitudinal studies on the tracking of serum lipid levels in children and adolescents. The only available data at the population level are from a study on baseline serum lipid levels in children.^16^,^17^

The purpose of this study was to track serum lipid levels over a four-year period in an urban population of schoolchildren.

## Methods

This prospective study was based on a survey of a cohort of schoolchildren aged 13 to 15 years. A similar study on subjects aged 17 to 19 years has been described in detail elsewhere.^18^ Our study began in 1999 with a cohort of 789 schoolchildren. Four years later this group was resurveyed and a further 452 adolescent were recruited to the study.

Data were collected anonymously using a self-administered questionnaire during a class session, with the collaboration of teachers and parents. All participants completed a questionnaire on their family history of cardiovascular disease, such as hypertension, and lifestyle characteristics including smoking, usual physical activity and dietary intake.

Research technicians recorded body weight to the nearest 0.1 kg using a standard beam balance scale with subjects barefoot and wearing light indoor clothing. Body height was recorded to the nearest 0.5 cm. Body mass index (BMI) was defined according to the Cole criterion.^19^

Taking into account the risk of bias due to observation, we opted for the electronic system to measure blood pressure. The reproducibility of measurements and the precision of this device has been demonstrated.^20^,^21^ After a 10-minute rest, we measured blood pressure on the right arm in the seated position, using an appropriate cuff size. We measured the blood pressure again after a 15-minute rest and the average was used in the analysis.

Participating children had to fast for 12 hours before blood was taken. Breakfast was served thereafter. A trained nurse with paediatric experience took the blood samples. Five millilitres of blood was collected in a tube containing 1 mg/ml EDTA and rapidly centrifuged. Plasma levels of total cholesterol (TC), low-density lipoprotein (LDL) cholesterol, high-density lipoprotein (HDL) cholesterol and triglycerides were measured and expressed as mmol/l. According to the American criteria for a paediatric population,^22^ we defined hypercholesterolaemia as ≥ 5.2 mmol/l, hypertriglyceridaemia as > 1.14 mmol/l, hyper-LDL cholesterol as > 3.4 mmol/l and hypo-HDL-C as < 0.9 mmol/l.

## Statistical analysis

The Student’s *t*-test and chi-squared test were used to analyse differences between groups in continuous and categorical variables. Tracking of lipid levels was evaluated using different statistical methods. Spearman product-moment correlations were calculated between the schoolchildren’s lipid levels measured at the ages of 13 to 15 and 17 to 19 years. The main effects on the lipid levels at 17 to 19 years were evaluated using a logistic regression model. The regression model for the school-children’s lipid levels at 17 to 19 years included lipid levels at 13 to 15 years, change in body mass index (∆BMI), gender, systolic blood pressure (SBP) and smoking. All statistical analysis was conducted using the statistical package SPSS 9.0. Statistical significance was set at *p* < 0.05.

Because of the young age of the target population, this investigation was undertaken with caution and with respect for the rights and integrity of people. We asked for authorisation from the Ministry of National Education, from teachers, the principals of schools and the parents of the selected children. Parents were free to refuse their child’s participation.

## Results

A total of 789 children were examined at baseline in 1999. Approximately 57.3% (452 children) were re-examined in 2003. Slightly more girls (*n* = 253) than boys (*n* = 199) were re-examined.

Selected follow-up characteristics of the cohort study are presented in Table 1. Significant differences between boys and girls were found in the means of TC, HDL-C, LDL-C, diastolic blood pressure (DBP) and ∆BMI values, and amount of smoking, hyperglycaemia and sedentary lifestyle.

**Table 1:** Follow-Up Parameters Of The Study Population According To Gender

	*Boys (n = 199)*	*Girls (n = 253)*	*p*
Mean of total cholesterol	4.05 ± 0.70	4.29 ± 0.78	0.001
Mean of triglycerides	0.92 ± 0.33	0.94 ± 0.41	0.664
Mean of HDL-C	1.57 ± 0.31	1.63 ± 0.28	0.028
Mean of LD-C	2.05 ± 0.58	2.22 ± 0.66	0.050
Mean of SBP	121.34 ± 19.00	121.10 ± 11.32	0.873
Mean of DBP	68.82 ± 11.05	72.12 ± 9.42	0.001
Mean of ∆BMI	2.89 ± 2.37	1.57 ± 2.14	< 10^-3^
% smoking	5.5	0.4	0.001
% hyperglycaemia	4	0.8	0.021
% sedentarity	4	4.7	0.711

**Fig. 1.**
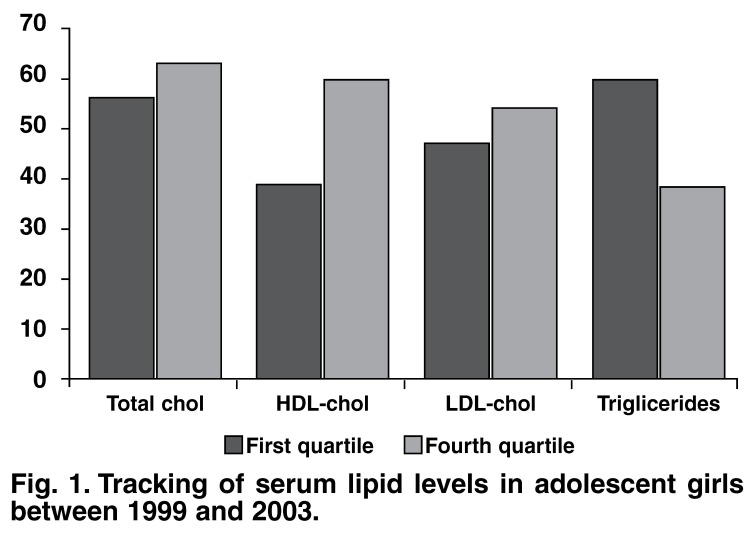
Tracking of serum lipid levels in adolescent girls between 1999 and 2003.

**Fig. 2.**
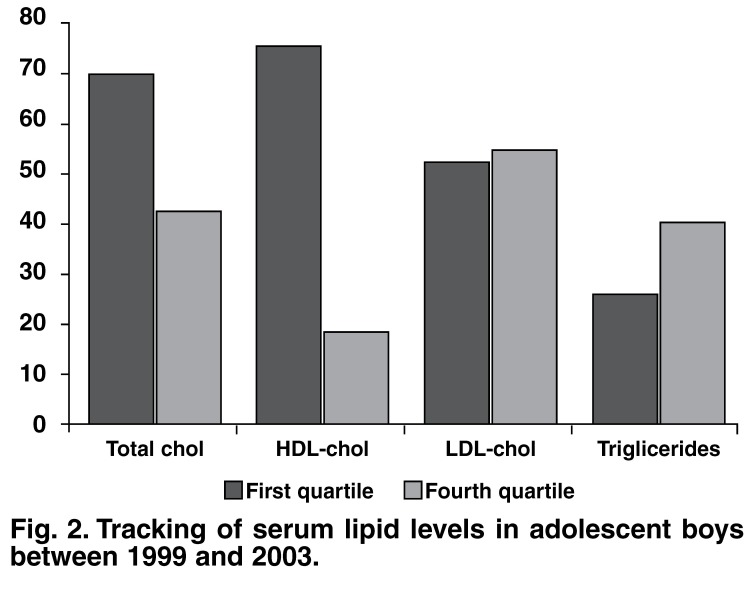
Tracking of serum lipid levels in adolescent boys between 1999 and 2003.

Figs 1 and 2 show the proportion of children in the highest and lowest gender-specific quartiles in 2003. The proportion of boys initially in the extreme quartile for TC, LDL cholesterol and trigliceride levels was 42.5, 54.8 and 40.4%, respectively. Similarly, for girls in the extreme quartile it was 62.7, 53.8 and 38.2%, respectively. Four years later, both the boys and girls were still in the extreme quartile for these parameters. The percentage of boys versus girls in the first quartile for HDL cholesterol values was 75.8 and 38.5%, respectively (*p* < 0.001).

Another measure of tracking is the correlation of serum lipid and lipoprotein levels taken at two time points. In Table 2 the correlation coefficients are shown for these parameters measured four years apart. All correlation coefficients were statistically significant at the *p* = 0.001 level. The independent variables included the baseline (1999) lipid level, ΔBMI, sedentary lifestyle, smoking status, glycaemia > 6.2 mmol/l, SBP and DBP.

**Table 2:** Correlation Matrices Of The Lipid Parameters Between 1999 And 2003

	*Total cholesterol*	*HDL-C*	*LDL-C*	*Triglycerides*
Total cholesterol	0.604***	0.287***	0.512***	0.122*
HDL-C	0.233***	0.601***	0.024	–0.161**
LDL-C	0.571***	0.096*	0.571***	0.114
Triglycerides	0.096*	–0.133*	0.094	0.377***

***p < 10^-3^; **p < 0.0011; *p < 0.00.

The best predictor of follow-up level for each of the serum lipoprotein cholesterol fractions was the corresponding baseline level (Table 3). Interestingly, the next best predictor in most of the groups was ΔBMI and smoking status (Table 3). The total amount of variability in the follow-up lipid levels explained by the baseline study variables ranged from 31.1 to 44.9%.

**Table 3:** Predictors Of Serum Lipid Levels By Gender After Four Years Of Follow Up From Baseline

	*Total cholesterol*	*HDL-C*	*Triglycerides*	*LDL-C*
Boys	Baseline total cholesterol	Basline HDL-C	Baseline triglycerides	Baseline LDL-C
	SBP	Glycaemia > 6.2 mmol/l	Smoking	Smoking
	Smoking	Sedentary lifestyle	∆BMI	∆BMI
	∆BMI			Glycaemia > 6.2 mmol/l
				SBP
				DBP
	*r^2^*= 0.402	*r^2^*= 0.349	*r^2^*= 0.311	*r^2^*= 0.391
Girls	Baseline total cholesterol	Basline HDL-C	Baseline triglycerides	Baseline LDL-C
	SBP	Glycaemia > 6.2 mmol/l	Smoking	Smoking
	Smoking	Sedentary lifestyle	∆BMI	∆BMI
	∆BMI			Glycaemia > 6.2 mmol/l
				SBP
				DBP
	*r^2^*= 0.435	*r^2^*= 0.435	*r^2^*= 0.435	*r^2^*= 0.435

## Discussion

In this study, we presented tracking coefficients and predictors of total cholesterol, HDL cholesterol, LDL cholesterol and triglyceride levels in a population-based cohort study over a period of four years. The percentage of boys initially in the extreme quartile for TC, LDL cholesterol and triglicerides was 42.5, 54.8 and 40.4%, respectively. Similarly, for girls in the extreme quartile it was 62.7, 53.8 and 38.2%, respectively. The correlation coefficients for serum lipids ranged from 0.60 to 0.37. The best predictor of follow-up level for each of the serum lipoprotein cholesterol fractions was the corresponding baseline level. The next best predictor in most of the groups was ΔBMI and smoking status.

The highest level of stability in the extreme quartile was 54.8% for LDL cholesterol levels in the boys and 62.7% for LDL cholesterol levels in the girls. Several previous studies among adolescents have shown the constancy of high lipid and lipoprotein levels.^23^,^24^ In the CATCH study,^25^ Kelder *et al*. revealed that 54 to 55% of students remained in quintile five for serum lipid levels four years later. In the Cardiovascular Risk in Young Finns study,^26^ approximately 50% of subjects who initially were in the extreme quintiles for total cholesterol, LDL cholesterol and HDL cholesterol levels were in the same quintiles after 12 years. In fact, in the Bogalusa heart study, approximately 50% of children who had total cholesterol levels above the 75th percentile at baseline still had this 12 years later.^27^ The Young Finns study^28^ demonstrated that 60 and 55% of subjects initially in the extreme quintiles for total cholesterol, LDL and HDL cholesterol remained there after three and six years, respectively.

The correlations we observed over time are in agreement with various other studies.^12^,^28^ In the Busselton study,^29^ a 27-year longitudinal study, the age- and survey year-adjusted Pearson’s correlation coefficients ranged from 0.35 to 0.55, and all were statistically significant. In the Bogalusa heart study,^27^ Larry *et al*. observed correlation coefficients for total cholesterol levels measured 12 years apart ranging from 0.38 to 0.66. In the Amsterdam Growth and Health study,^30^ the correlation was significant, with a coefficient of 0.71 for total cholesterol and 0.51 to 0.65 for HDL cholesterol levels. Similar results were shown by Porkka *et al*.^26^ in 12 years of tracking of serum lipid levels. In fact, correlation coefficients were higher for TC, LDL-C and HDL-C (0.48–0.59) than for triglyceride levels (0.33–0.37).

The Cardiovascular Risk in Young Finns study^27^ observed that the initial childhood or adolescent serum lipid value was the most significant of each adult serum lipid variable. The total amount of variability in the follow-up lipid levels explained by the baseline study variables ranged from 4 to 41%.^26^ Webber *et al.* noticed that percentage variability was lower for triglyceride than for cholesterol levels and ranged from 6 to 41%.

## Conclusion

Serum lipids show constancy from childhood and adolescence to adulthood, and therefore can be used as markers for the potential risk of developing premature atherosclerosis. Prevention of coronary heart diseases must begin early on in childhood, and should be driven by health education towards making healthy lifestyle choices.
